# From intermittent antibiotic point prevalence surveys to quality improvement: experience in Scottish hospitals

**DOI:** 10.1186/2047-2994-2-3

**Published:** 2013-01-15

**Authors:** William Malcolm, Dilip Nathwani, Peter Davey, Tracey Cromwell, Andrea Patton, Jacqueline Reilly, Shona Cairns, Marion Bennie

**Affiliations:** 1 NHS National Services Scotland, Health Protection Scotland, Meridian Court, 5 Cadogan Street, Glasgow, Scotland, G2 6QE, UK; 2 Ninewells Hospital & Medical School, Dundee, Scotland, DD19SY, UK; 3 Population Health Sciences Division, Medical Research Institute, Dundee, Scotland, DD2 4BF, UK; 4 NHS National Services Scotland, Information Services Division, Gyle Square 1 South Gyle Crescent, Edinburgh, Scotland, EH12 9EB, UK; 5 Healthcare Improvement Scotland, Gyle Square 1 South Gyle Crescent, Edinburgh, Scotland, EH12 9EB, UK; 6 Strathclyde Institute of Pharmacy and Biomedical Sciences, University of Strathclyde, Taylor Street, Glasgow, Scotland, G4 ONR, UK

**Keywords:** Antimicrobial stewardship, Quality improvement, Prescribing indicators, Point prevalence survey, Antibiotic, Hospital prescribing, Surgical prophylaxis

## Abstract

**Background:**

In 2008, the Scottish Antimicrobial Prescribing Group (SAPG) was established to coordinate a national antimicrobial stewardship programme. In 2009 SAPG led participation in a European point prevalence survey (PPS) of hospital antibiotic use. We describe how SAPG used this baseline PPS as the foundation for implementation of measures for improvement in antibiotic prescribing.

**Methods:**

In 2009 data for the baseline PPS were collected in accordance with the European Surveillance of Antimicrobial Consumption [ESAC] protocol. This informed the development of two quality prescribing indicators: compliance with antibiotic policy in acute admission units and duration of surgical prophylaxis. From December 2009 clinicians collected these data on a monthly basis. The prescribing indicators were reviewed and further modified in March 2011. Data for the follow up PPS in September 2011 were collected as part of a national PPS of healthcare associated infection and antimicrobial use developed using ECDC protocols.

**Results:**

In the baseline PPS data were collected in 22 (56%) acute hospitals. The frequency of recording the reason for treatment in medical notes was similar in Scotland (75.9%) and Europe (75.7%). Compliance with policy (81.0%) was also similar to Europe (82.5%) but duration of surgical prophylaxis <24hr (68.6%), was higher than in Europe (48.1%, OR: 0.41, p<0.001). Following the development and implementation of the prescribing indicators monthly measurement and data feedback in admission units illustrated improvement in indication documented of ≥90% and compliance with antibiotic prescribing policy increasing from 76% to 90%. The initial prescribing indicator in surgical prophylaxis was less successful in providing consistent national data as there was local discretion on which procedures to include. Following a review and a focus on colorectal surgery the mean proportion receiving single dose prophylaxis exceeded the target of 95% and the mean proportion compliant with policy was 83%. In the follow up PPS of 2011 indication documented (86.8%) and policy compliant (82.8%) were higher than in baseline PPS.

**Conclusions:**

The baseline PPS identified priorities for quality improvement. SAPG has demonstrated that implementation of regularly reviewed national prescribing indicators, acceptable to clinicians, implemented through regular systematic measurement can drive improvement in quality of antibiotic use in key clinical areas. However, our data also show that the ESAC PPS method may underestimate the proportion of surgical prophylaxis with duration <24hr.

## Background

The importance of an integrated programme to reduce Healthcare Associated Infection (HAI) is recognised by the Scottish Government [1]. In 2008 the Scottish Government funded the establishment of the Scottish Antimicrobial Prescribing Group (SAPG) to coordinate the delivery of a national antimicrobial stewardship programme to enhance the quality of antimicrobial prescribing, timely and appropriate management of infection and the reduction of collateral damage from unnecessary antibiotic use [2][3]. SAPG is a national clinical multidisciplinary forum with representation from all 14 National Health Service (NHS) boards in Scotland as well as key stakeholders involved in infection prevention, surveillance and quality improvement [2]. SAPG coordinates a network of antimicrobial management teams (AMTs) which include a lead doctor and antimicrobial pharmacist [4]. The AMTs are responsible for delivery of antimicrobial stewardship in NHS boards. In 2008 the Scottish Government provided funding to enable the appointment of antimicrobial pharmacists in all NHS boards [5].

Point prevalence surveys (PPS) have been used successfully to monitor antimicrobial use in hospitals [6][7]. The ESAC programme developed a standardised data collection technique to support monitoring of trends in prescribing and identification of priorities for quality improvement. The ESAC methodology can be successfully applied at national level [8].

In 2007 a prevalence survey of HAI in Scottish hospitals demonstrated 32.1% of patients were prescribed antimicrobials; whilst this established a baseline of the burden of prescribing it contained no information on measures of prescribing quality [9]. In 2009 SAPG coordinated a national Scottish acute hospital PPS in collaboration with ESAC. The objectives were to identify areas of variable or poor practice with a view to developing prescribing indicators for quality improvement, establish the national baseline for these indicators and comparison against European data. After establishing the baseline, monthly measurement of prescribing indicators in key clinical areas was implemented in December 2009 to drive improvement in the quality of hospital prescribing of antimicrobials. The national prescribing indicators were reviewed and modified in March 2011. A follow up national PPS was undertaken in September 2011.

We report on the experience and outcome of how SAPG engineered this move from infrequent hospital wide PPS to a structured national programme of focused quality improvement in use of antimicrobials.

## Methods

### Timeline

**Figure 1:**
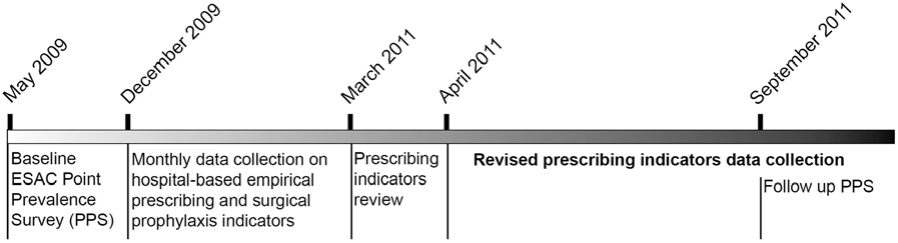
Time line showing progress from Point Prevalence Survey to Continuous Quality improvement.

The timeline (Figure 1) illustrates when the various steps in the work were undertaken.

### Baseline PPS (May 2009)

We invited all NHS board AMTs to participate in the ESAC 2009 PPS and recommended that a minimum of one acute hospital should be recruited in each NHS board. To encourage recruitment we wrote to senior clinicians including Medical Directors and Directors of Pharmacy to obtain their support. Data were collected in each hospital over a maximum two week period between 1^st^ May 2009 and 26^th^ June 2009. Data were collected in accordance with the ESAC PPS 2009 protocol by multidisciplinary teams including pharmacists, microbiologists and infectious disease specialists [8]. We developed support materials to complement the ESAC protocol for lead investigators in AMTs. Data were entered into WebPPS, a web application developed by ESAC for data entry, analysis and reporting [8]. Data were mainly inserted centrally except in three NHS boards where entry was undertaken locally by the AMTs. These data were aggregated and analysed using SPSS statistical software (version 17) following permission from NHS boards and compared with European data provided by ESAC.

### Prescribing indicators as national targets (December 2009)

In consultation with clinicians SAPG agreed two national prescribing indicators to drive improvements in the quality of hospital prescribing. These were disseminated to NHS boards as part of a revised national surveillance framework from Scottish Government [10]. These indicators were:

*Hospital-based empirical prescribing*: choice of antibiotic prescribed is compliant with the local antimicrobial policy (policy compliant) and the rationale for treatment is recorded in the clinical case note (indication documented) in ≥95% of sampled cases.

*Surgical antibiotic prophylaxis*: duration of surgical antibiotic prophylaxis is <24hr (duration <24hr) and choice of antibiotic prescribed is compliant with local antimicrobial prescribing policy (policy compliant) in ≥95% of sampled cases.

Empirical prescribing measures were collected by local clinicians in a sample of 20 patients per month in acute (medical and surgical) admissions units. Data were collected primarily by antimicrobial pharmacists but in some NHS boards data were collected by junior doctors/nursing staff. Patients in other inpatient facilities were not audited. Data on surgical prophylaxis were collected in a sample of 20 patients per month in at least two common surgical procedures, identified locally by each NHS board. The sampling technique was either five patients each week or 20 patients in a single week. To minimise bias the day of the week data were collected was rotated. The data source was the patient’s medical notes and drug prescription chart. Data were collected in accordance with a simple protocol developed by SAPG and distributed to AMTs. No specific training on data collection was provided. If the indication for treatment was not documented then compliance with policy was recorded as non-compliant. If an indication was not covered by the local antibiotic policy the case was excluded. Local clinicians entered data onto a SAPG extranet, a secure website for supporting quality improvement projects provided by the Institute for Healthcare Improvement [11]. The system enabled immediate feedback in the form of standardised run charts for clinicians which facilitated data sharing between AMTs. Data were aggregated to provide national compliance with prescribing indicators for feedback to the 14 NHS boards in quarterly reports, which compared their performance with other boards. Training was provided to AMTs on how to use the Extranet. No additional funding or resource was allocated to AMTs to participate in the quality improvement initiative.

### Review of Prescribing Indicators as National Targets (March 2011)

Following discussion with AMTs the prescribing indicators were reviewed in March 2011 to refine the methodology for data collection, improve consistency and enable the data to further drive quality improvement. From April 2011, AMTs continued to collect empirical prescribing measures in acute admission units but data were reported separately for medical and surgical admissions. The sampling strategy and data sources remained the same. The methodology was refined to allow prescriptions that deviated from policy to be marked as compliant if a clearly justified reason for deviating from policy was documented in the patient’s notes. If indication for treatment was not documented in the patient’s notes the case was marked as non-compliant in ‘Indication documented’ and excluded from the policy compliance measure. This meant that ‘Policy Compliant’ indicated the percentage of patients whose antibiotic treatment deviated from policy with no documented reason. We did not assess inter-rater reliability in this study but the definition of policy compliance was adapted from a research study on skin and soft tissue infection in one of the participating hospitals. The inter-rater reliability for policy compliance in the previous study was 85%, two way kappa =0.61 (95% CI 0.41-0.80, p<0.01) indicating good agreement [12]. In addition to routine data collection, SAPG asked AMTs to detail up to five cases of non-compliance per month. This information was fed back to prescribers locally to improve prescribing practice and also to SAPG to identify common themes that could help shape national educational solutions as well as quality improvement projects.

For surgical prophylaxis, from April 2011, AMTs were asked to focus data collection on a single surgical procedure to achieve a national consistency. The ‘policy compliant’ measure was retained but the duration measure was changed from duration <24hr to single dose prophylaxis. Elective colorectal surgery, a high burden disease, was chosen as a procedure where a single prophylactic dose was recommended [13] and where local prescribing policies contained similar recommendations on choice of prophylactic antimicrobial but where compliance was reportedly low.

### Follow up PPS (September 2011)

All acute hospitals were included in the Scottish Government’s 2011 national PPS of HAI and antimicrobial prescribing developed and implemented by Health Protection Scotland (HPS). Data were collected in accordance with the Scottish protocol for data collection [14] which was developed using the European Centre for Disease Prevention and Control protocol for PPS (which encompassed the previous ESAC protocol used for our baseline PPS) [15]. Data were collected between 1^st^ September 2011 and 31^st^ October 2011 by a collaborative team from local Infection Control Teams and AMTs. Data were collected on Teleform® paper forms, one form per ward and one form per patient; this was sent securely to HPS by post adhering to data protection and confidentiality guidelines. Each form was scanned and verified by data entry staff and imported into a SQL Server database®. The data were quality checked using a Microsoft Access® database and Stata Version 9® prior to analysis.

### Statistical Analysis

The baseline PPS survey included 31 hospitals from Scotland. The results presented in this paper are for acute hospitals only (n=22). The European data were from hospitals submitted to ESAC PPS as of January 2010. An Odds Ratio was calculated to compare the baseline PPS results for Scotland against results for the whole of Europe. A p-value <0.05 was considered to be statistically significant. In the follow up PPS data were submitted from 42 acute hospitals in Scotland. For the quality improvement indicators, percentage compliance was calculated using number of cases compliant with measure / number of cases tested x100.

## Results

### Baseline PPS (May 2009)

#### Hospital overview

Data were collected in 22 (56%) acute hospitals, incorporating 8,253 (60%) acute beds in Scotland and covering 13 NHS boards. These included teaching hospitals providing a full range of clinical services to general hospitals with a mixture of medical and surgical specialties. In participating hospitals bed numbers ranged from 43 to 879. Only one small island NHS board with a single acute hospital (109 beds) did not participate.

#### Prescribing overview

The results are summarised in Table 1. In total 7,573 patients in Scotland were surveyed. Overall 30.2% of Scottish patients were prescribed an antimicrobial, which was similar to Europe (29%). The frequency of recording the reason for treatment in medical notes was similar in Scotland (75.9%) and Europe (75.7%). Compliance with policy (excluding cases where compliance was not assessable or where no information was available) in Scotland was 81.0%, similar to Europe (82.5%). Surgical prophylaxis accounted for 8.9% of total antimicrobial use in Scotland. In Scotland the duration of surgical prophylaxis was <24hr in 68.6%, significantly higher than in Europe (48.1%, OR: 0.41, p<0.001).

**Table 1:** Overview of prescribing from baseline PPS (May 2009) and follow up PPS (September 2011)

**Measure**	**Baseline PPS (May 2009)**			**Follow up PPS** **(Sept 2011)**
	**Scotland acute hospitals**	**Europe**	**Odds ratio (p value)**	**Scotland acute hospitals**
Number of patients surveyed	7,573	73,060		11,604
Number of patients (%) prescribed antimicrobials	2,289 (30.2%)	21,197 (29.0%)	1.06 (0.03)	3,728 (32.3%)
Number of patients (%) prescribed single antimicrobial	1,432 (62.6%)	14,403 (67.9%)	0.79 (<0.001)	2,268 (60.8%)
Number of prescriptions (%) for parenteral antimicrobials	1,731 (51.8%)	17,947 (60.5%)	0.7 (<0.001)	2,147 (47.8%)
Number of prescriptions (%) with indication recorded in notes	2,538 (75.9%)	22,456 (75.7%)	1.01 (0.78)	3,811 (86.8%)
Number of prescriptions (%) compliant with local policy	1939 (81.0%)	17,223 (82.5%)	0.90 (0.06)	2,245 (82.8%)
Number of surgical prophylaxis prescriptions (%) with duration single dose	146 (49.3%)	927 (27.0%)	2.92 (<0.001)	287 (59.5%)
Number of surgical prophylaxis prescriptions (%) with Duration = 1 day	57 (19.3%)	723 (21.1%)	0.85 (0.27)	81 (16.8%)
Number of surgical prophylaxis prescriptions (%) with duration >1 day	93 (31.4%)	1783 (51.9%)	0.41 (<0.001)	114 (23.7%)

### Prescribing indicators as national targets (December 2009)

#### Hospital-based empirical prescribing

**Figure 2:**
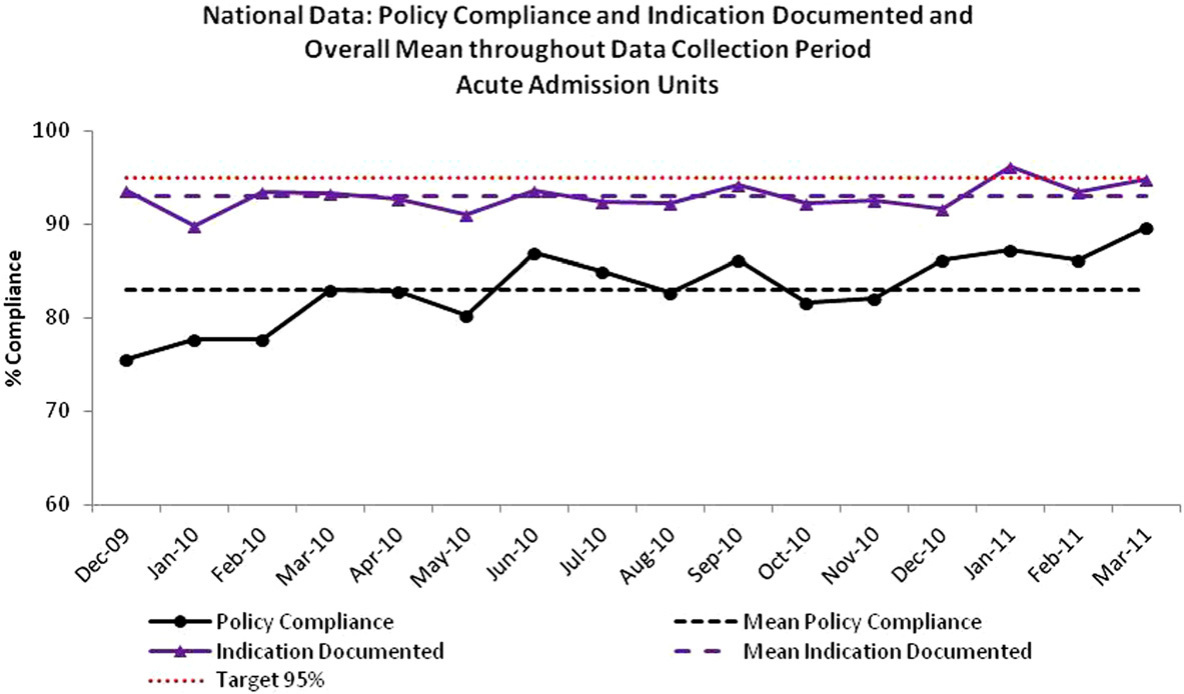
Hospital empiric prescribing: National compliance with Indication Documented and Policy Compliant (antibiotic choice) and overall mean, December 2009-March 2011.

Between December 2009 and March 2011 data for 10,617 patients in 49 medical and surgical admission units were submitted to the SAPG Extranet. During this data collection period there was a steady increase in the number of units submitting data. Compliance with indicators varied between NHS boards and between hospitals within each NHS board. The aggregated national results illustrate compliance with ‘Indication Documented’ was ≥90% throughout the data collection period with overall mean of 93% and ‘Policy Compliance’ increased from 76% to 90% during the data collection period with an overall mean of 83%. (Figure 2). There were no NHS boards where the intervention did not result in some improvement. One hospital achieved 100% compliance with both measures throughout the data collection period. In this instance there was a team of three acute medical consultants and also acute medical registers who were engaged in the quality control initiatives and constantly reinforced their importance to the staff.

#### Surgical prophylaxis

Between December 2009 and March 2011 surgical prophylaxis data for 7,344 patients were submitted to the SAPG Extranet by 10 NHS boards across six surgical specialties; orthopaedics (2360); cardiac/cardiothoracic (596); general surgery including gastrointestinal and colorectal (2747); obstetrics/ gynaecology (791); vascular (412) and urology (438). Mean performance across the data collection period with ‘Duration <24hr’ and ‘Policy Compliance’ by surgical specialty are shown in Table 2.

**Table 2:** Mean (min, max) compliance with surgical prophylaxis measures by surgical specialty for surgical prophylaxis indicator December 2009-March 2011

	**Duration <24h Mean (Min, Max)**	**Policy Compliance Mean (Min, Max)**
Cardiac / Cardiothoracic	96% (88%, 100%)	95% (85%, 100%)
General Surgery (including GI & Colorectal)	91% (84%, 100%)	68% (50%, 79%)
Obstetrics / Gynaecology	98% (93%, 100%)	87% (72%, 96%)
Orthopaedics	98% (94%, 100%)	93% (85%, 97%)
Urology	76% (70%, 100%)	79% (68%, 100%)
Vascular	96% (80%, 100%)	93% (50%, 100%)

### Review of Prescribing Indicators as National Targets (March 2011)

#### Hospital-based empirical prescribing

**Figure 3:**
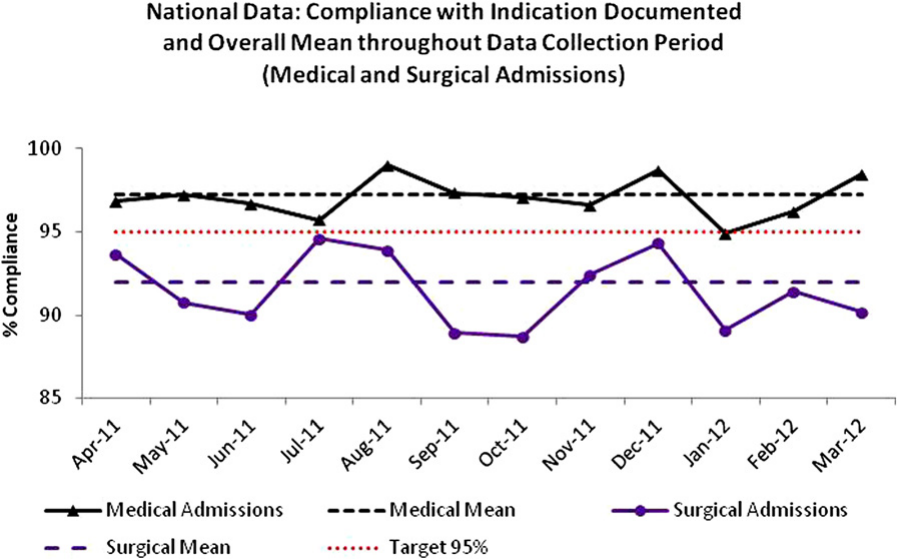
Hospital empiric prescribing: National compliance with Indication Documented in medical and surgical admissions and overall mean, April 2011-March 2012.

**Figure 4:**
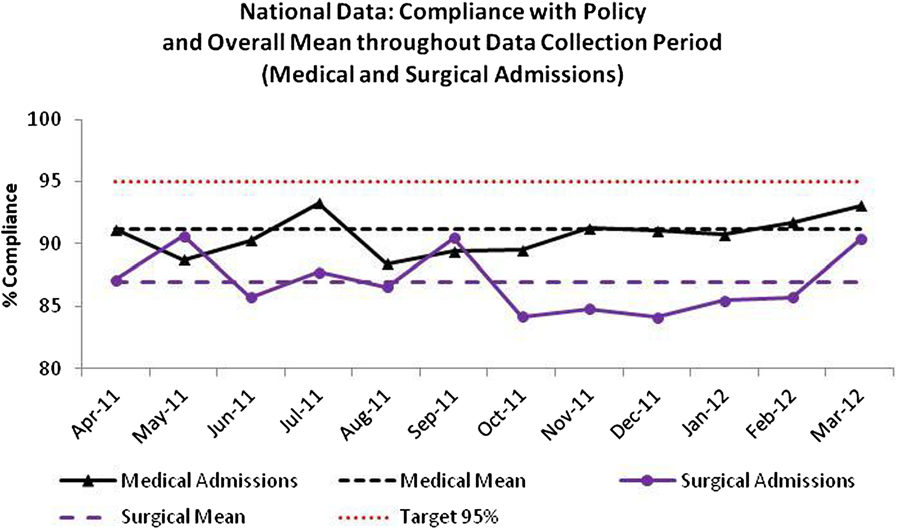
Hospital empiric prescribing: National Policy Compliance in medical and surgical admissions and overall mean, April 2011-March 2012.

Empirical prescribing data from medical admissions units were submitted to the SAPG Extranet from all 14 NHS boards between April 2011 and March 2012. Compliance with ‘Indication Documented’ was assessed in 5152 patients and overall the national mean was 97% (Figure 3). Compliance with antibiotic choice was assessed in 5000 patients and overall national mean was 91% (Figure 4). Surgical admissions unit data were submitted to the SAPG Extranet from 13 NHS boards between April 2011 and March 2012. ‘Indication Documented’ was assessed in 3031 patients. Compliance with indicators varied between NHS boards and between hospitals within boards. Overall, the national mean compliance was 92% (Figure 3). Compliance with policy was assessed in 2779 patients and the overall national mean was 87% (Figure 4). In addition to presenting monthly results to AMTs and Government at national level each NHS board received a report detailing performance at a local level. As of March 2012 some NHS boards have been instructed to move to less frequent data collection (three monthly) as they have demonstrated sustained improvement [16] with the indicators allowing the board to focus improvements in other areas of the hospital.

#### Surgical prophylaxis

**Figure 5:**
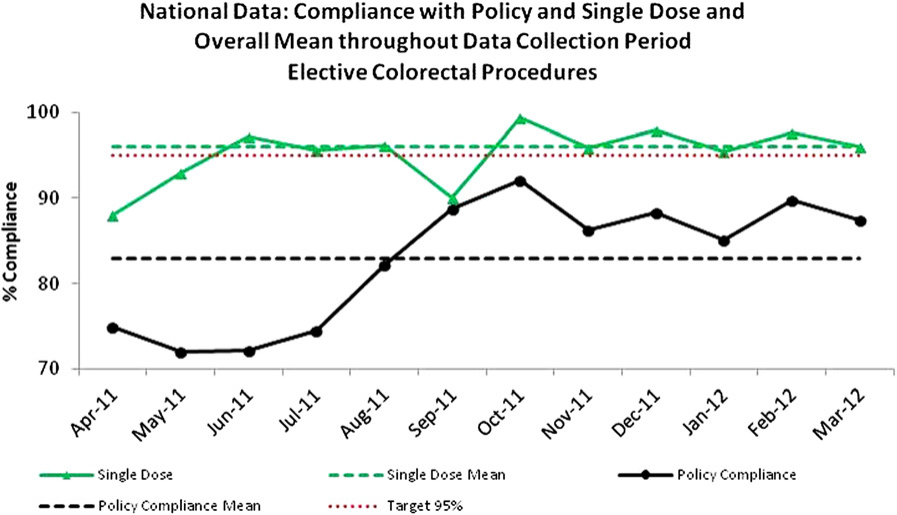
Surgical prophylaxis: National compliance with Single dose and Policy Compliance in elective colorectal procedures and overall mean, April 2011-March 2012.

Surgical prophylaxis data were collected on 2258 elective colorectal procedures between April 2011 and March 2012. Overall national mean compliance with ‘Single Dose’ was 96% and 83% for ‘Policy Compliant’ (Figure 5).

### Follow up PPS (September 2011)

In total 11,604 patients were surveyed across all (42) acute hospitals in Scotland. A total of 3,728 (32.3%) patients were receiving antimicrobials at the time of the survey. The results for key measures of prescribing quality were: compliance with policy (82.8%); reason for treatment documented in medical notes (86.8%); duration of surgical prophylaxis <24hr (76.3%) (Table 1)

## Discussion

This evaluation describes how SAPG has coordinated a hospital wide PPS and built on this to implement regular measurement and feedback of nationally agreed prescribing indicators that have driven improvements in antimicrobial prescribing in Scottish acute hospitals.

SAPG coordinated the participation of 56% of acute hospitals in Scotland in the ESAC PPS 2009 (baseline PPS). This level of participation was due to a combination of national leadership [from SAPG] and local engagement from AMTs and clinicians. In the follow up PPS data were collected in all acute hospitals in Scotland following a Scottish Government instruction to all NHS boards to participate.

Previous data from the 2006 ESAC PPS [17], and a small Scottish survey in 2007 [18], had indicated compliance with prescribing policy and antimicrobial use for surgical prophylaxis were areas of variable practice in Scotland. The baseline PPS data proved valuable in identifying key measures intended to drive improvement in the quality of antimicrobial use in hospitals; poor documentation of indication for treatment and compliance with local prescribing policy remained features of poor practice in empirical prescribing. Furthermore prolonged duration of surgical prophylaxis was also confirmed as an area where improvement was required.

Documenting the reason why an antimicrobial has been prescribed in medical notes is recommended in Scotland as essential for good clinical practice [4]. It ensures communication of diagnosis between clinical teams and supports review of treatment. It was therefore disappointing that no indication was documented in 24.1% cases, and although similar to Europe, SAPG regarded this level as unacceptable and an important target for improvement. All AMTs have produced guidelines on empirical treatment of commonly encountered infections based on advice from SAPG issued in 2008 [19]. The baseline PPS revealed compliance with local policy of 81.0% (excluding cases where compliance was not assessable or where no information was available) confirming the need for improvement. Although the proportion of surgical prophylaxis with a duration of <24hr was greater than the European average, in 31.4% of cases it exceeded 24hr in the baseline PPS. The Scottish Intercollegiate Guideline Network (SIGN) guideline on antibiotic prophylaxis in surgery recommended a single dose of an appropriate antimicrobial is required for the majority of surgical procedures [13]. Unnecessarily prolonged surgical prophylaxis contributes to selection pressure for antimicrobial resistance [20] and associated risks such as *Clostridium difficile* infection (CDI) [21].

Disadvantages of hospital wide PPS include the labour required to collect and input data, their infrequent nature and the requirement for centralised data input resulting in a delay in reports being available for participating hospitals. When available the results of the baseline PPS were fed back to clinical teams by AMTs to encourage better local prescribing although their impact was probably reduced as the data were not recent. To overcome this and provide a further stimulus SAPG introduced regular systematic measurement of quality using national prescribing indicators in key clinical areas to promote improvement. The indicators chosen underpinned a national target for reduction in CDI [10]. By example compliance with local antimicrobial policy promoted agents less likely to lead to CDI and reducing excessively prolonged surgical prophylaxis would also reduce CDI [20][21]. We believe linking the national prescribing indicators to a key clinical area of concern combined with the support of Government to include the indicators as an integral component of national initiatives for patient safety and quality improvement was pivotal to their successful introduction and adoption by clinicians [22].

Establishing a culture of measurement and clinician feedback is an effective stewardship strategy [23], and using this approach the hospital empiric prescribing indicator has been successful in providing early evidence of improvement in medical and surgical admission units although this yet not consistent and reliable across all NHS boards. Our intervention was audit and feedback and three recent meta-analyses have used behavioural theories to synthesise evidence from audit and feedback studies in order to identify intervention components that may enhance effectiveness. One review used Feedback Intervention Theory [24] and two used Control Theory [25]. All three reviews suggested that the effectiveness of audit and feedback is enhanced by setting a target or behavioural goal, which was a component of our intervention. In addition effectiveness was improved in the Feedback Intervention Theory by providing specific, frequent and written suggestions for improvement [24]. One Control Theory review found that insufficient studies reported on use of action plans to allow reliable statistical analysis [25] However, a larger and more recent review indicated that feedback may be more effective when baseline performance is low, the source is a supervisor or colleague, it is provided more than once, it is delivered in both verbal and written formats, and when it includes both explicit targets and an action plan [26], results that are consistent with Control Theory [25]. We asked all AMTs to collect up to five examples of non-compliance per month and used this information both locally and nationally to identify common themes that could help shape national educational solutions. However, we did not attempt to standardize the way that this information was fed back to clinical teams (e.g. in writing *versus* verbally) or to what extent the information was used for action planning. We will consider the feasibility of more explicit application of these theoretical frameworks to future interventions and to understanding variation in the success of our current interventions.

We suggest the reported difference between the baseline PPS and the subsequent prescribing indicators may be due in part to the PPS evaluating prescribing across the whole hospital rather than only in medical and surgical admission units where the prescribing indicators were applied. Consequently there may have been a greater emphasis on improvement of prescribing in admission units compared with other parts of hospitals. Indeed, improving the quality of prescribing and antibiotic review in continuing care inpatient wards has been identified as an area of priority for SAPG in the future. In the follow up PPS there were higher levels of indication documented and policy compliance than observed in the baseline survey. The increase is welcome but remains below the targets used in the national prescribing indicators and confirms the need to introduce improvement initiatives in other inpatient departments.

For sustainable clinical engagement we believe it is important that national prescribing indicators are open to review and seen as drivers for improving clinical outcomes as opposed to being viewed as either punitive or restrictive measures. Our review in March 2011 showed that although compliance with the hospital empiric prescribing indicator measures had improved AMTs indicated that monitoring and reporting acute medical and surgical admissions units separately would provide greater clarity. Data following the review illustrated a lower proportion of indication documented and policy compliance in surgical units and led to AMTs targeting improvement activity more closely with clinicians in surgical units.

The initial prescribing indicator for surgical prophylaxis was less successful in providing a consistent and homogenous national dataset as AMTs could select which surgical procedures to include. The review of the surgical prophylaxis indicator to focus on colorectal surgery from April 2011 has achieved a consistency of approach not possible when there was local discretion over which procedures to include. Nationally aggregated data for 12 months following the modification illustrate the proportion receiving single dose prophylaxis has exceeded the target of 95% and the policy complaint proportion has increased but remains below the target indicating that further improvement work is required. Although data for the initial prescribing indicator were collected in a number of surgical procedures the proportion with duration <24hr was much higher at the start of data collection than observed in the baseline PPS. It is possible that surgeons improved their practice after the baseline PPS but it is more plausible that the difference is due to the way doses for surgical prophylaxis were recorded and captured. In the baseline PPS only standard prescription charts were used to identify doses for prophylaxis. However, in routine clinical practice in Scotland prophylactic single doses for prophylaxis may be prescribed in the once only section of standard prescription charts, on theatre record sheets or on the fluid prescription chart. If only standard prescription charts are reviewed, then single doses prescribed on other records will not be captured with a resultant overestimation of the proportion of prolonged prophylaxis. This may require to be considered in reviewing the methodology used in the PPS. The follow-up PPS used all case notes, nursing notes and theatre records. In the follow up PPS there was a higher proportion of surgical prophylaxis with a duration of <24hr than in the baseline survey but it remains lower than observed in the national prescribing indicator dataset. These data indicate that improvement may still be required in procedures other than colorectal surgery.

We believe involvement of the prescribing and clinical community in collecting and feeding back these data was important. The improvement in the national prescribing indicators has only been possible through engagement with AMTs and the clinical community in Scotland. This has been challenging with surgeons, where discussions have centered on the choice of prophylaxis regimen. The move away from cephalosporins to narrower spectrum beta-lactams with or without aminoglycoside has met with some resistance, partly because the evidence based to support these regimens against traditional agents such as cefuroxime was not available. As part of ongoing dialogue with the surgical community, and other clinicians, SAPG has committed to measure unintended consequences of changes in prescribing policies including aminoglycoside and flucloxacillin related renal toxicity, increased surgical site infection and mortality. SAPG believe such balancing measures are critical in reassuring clinicians of the safety and effectiveness of our interventions.

There are some limitations to the methods used. In the PPS data were collected across hospitals with different specialties and case mix. This may influence the prevalence of antimicrobial use but quality measures such as recording in notes, compliance with policy and duration of surgical prophylaxis should be less affected. As numerous individuals collected baseline and prescribing indicator data the extent of inter-rater variation or observer bias is unknown. Although in the follow-up PPS inter-rater reliability was tested and found to indicate an excellent level of agreement between data collectors [27]. The improvements in the prescribing indicator data have not been statistically assessed. The sample size for prescribing indicators as national targets may have influenced the results i.e. with a recommended sample of 20 patients per month, to achieve the target of ≥95% compliance a score of ≥19/20 is required. When sample size was <20 patients adherence to policy and documenting indication had to be perfect to achieve the target.

## Conclusions

Our experience shows that hospital wide PPS conducted on an infrequent basis are valuable in identifying priorities for quality improvement and establishing their baseline. These priorities have informed the development of national prescribing indicators that are acceptable to clinicians and the infection community. Their measurement has been embedded, where possible, into routine clinical practice and is primarily used as a driver for local improvement but nationally they also provide information towards our goal of attainment of a national CDI target. We hope our methods and the lessons learnt will inform and encourage other healthcare systems to consider such methods \towards improving the quality of prescribing in hospitals.

## Competing interests

The Scottish Antimicrobial Prescribing Group is supported by funding from the Scottish Government Health Department. The authors declare that they have no competing interests.

## Authors’ contributions

WM, DN, PD, TC, AP and MB are members of the Scottish Antimicrobial Prescribing Group (SAPG) and have made substantial contributions to the design and delivery of SAPG work programme. WM was responsible for the co-ordination of Scottish hospitals participation in the baseline point prevalence survey. DN has been chair of SAPG since 2008. DN, PD and MB made substantial contributions to the development of prescribing indicators as national targets to drive quality improvement. TC was responsible for performing the analysis of the Scottish data from the national point prevalence survey. AP was responsible for the development and maintenance of the data management system for measuring and reporting the national prescribing indicators. JR was responsible for the implementation of the 2011 PPS. SC was responsible for analysis of data for the follow up point prevalence survey. All authors have read and approved the final manuscript.

## Authors’ information

WM is Pharmaceutical Adviser in NHS National Services Scotland with MSc Clinical Pharmacy and Master of Public Health.

DN is chair of SAPG and is a Consultant Physician and Honorary Professor of Infection, Ninewells Hospital and Medical School, Dundee, Scotland, UK.

PD is Medical School Lead for Clinical Quality Improvement, Population Health Sciences Division, Medical Research Institute, Dundee, Scotland, UK.

TC is an information analyst with MSc in applied statistics/business statistics.

AP is an information analyst with an MSc in applied statistics.

SC is an epidemiologist with BSc in immunology and pharmacology and MSc in epidemiology.

JR is Lead Consultant in Healthcare Associated Infection in NHS National Services Scotland and Professor of Healthcare Associated Infection. Caledonian University, Glasgow, Scotland, UK.

MB is Chief Pharmaceutical Adviser in NHS National Services Scotland and Professor of Pharmacy Practice, University of Strathclyde, Glasgow, Scotland, UK.
